# Application of In Vitro Digestion Models in the Evaluation of Dietary Supplements

**DOI:** 10.3390/foods13132135

**Published:** 2024-07-04

**Authors:** Justyna Ośko, Katarzyna Nasierowska, Małgorzata Grembecka

**Affiliations:** 1 Department of Bromatology, Faculty of Pharmacy, Medical University of Gdańsk, Gen. J. Hallera Avenue 107, 80-416 Gdańsk, Poland; 2 Student Scientific Circle, Department of Bromatology, Faculty of Pharmacy, Medical University of Gdańsk, Gen. J. Hallera Avenue 107, 80-416 Gdańsk, Poland; katarzyna.nasierowska@gumed.edu.pl

**Keywords:** dietary supplements, in vitro model, bioavailability, bioaccessibility

## Abstract

Nowadays, dietary supplements are a permanent part of our diet. Using various simulated in vitro digestive models, the bioavailability of dietary supplement ingredients has also been investigated. In most cases, static models are used instead of dynamic ones. This article focuses on the division of applications of in vitro methods, such as assessing the quality of dietary supplements (in chemical and pharmaceutical form), the impact of diet on the assessment of the bioavailability of product ingredients, the impact of supplement ingredients on the state of intestinal microflora, and the development of new products using various encapsulation methods. The review included publications from 2000 to 2024 showing the use of in vitro methods in dietary supplements containing polysaccharides, proteins, elements, vitamins, and bioactive substances, as well as probiotic and prebiotic products. The impact of components in dietary supplements on the human digestive tract and their degree of bioaccessibility were determined through the use of in vitro methods. The application of in vitro methods has also become an effective tool for designing new forms of dietary supplements in order to increase the availability and durability of labile ingredients in these products.

## 1. Introduction

Nowadays, a growing range of food products constitute dietary supplements. Due to the constant demand for such foods, many researchers have undertaken targeted studies to evaluate their composition and quality. However, information on the individual components’ content in dietary supplements (including adulteration with other undesirable substances) may be only a part of the information obtained from the analyses. The bioavailability/bioaccessibility of ingredients contained in dietary supplements is important information that could supplement the label of a given product. This could be helpful, for instance, when it comes to labeling dietary supplements as tailored products for particular ingredient deficiencies that arise on a case-by-case basis. The term “bioavailability” actually has many working definitions, which depend on the research area it concerns. In the context of food and nutrition science, this term refers to an ingested nutrient or compound that is released from the food matrix during digestion and is available for absorption in the small intestine or is biotransformed by the intestinal microflora [1]. According to the definition provided by Benito and Miller [2], it is also the portion of an ingredient or compound that the organism can actually use. This concept can be further divided into “bioaccessibility” and “bioactivity”. The expression “bioaccessibility” refers to the fraction of a compound released from the matrix in the gastrointestinal tract that is in an assimilable form and can be absorbed in the intestinal environment. “Bioaccessibility” encompasses all the processes involved in digesting food in order to convert it into potentially bioavailable material. However, it excludes absorption/assimilation by epithelial tissue and pre-systemic metabolism (both intestinal and hepatic) [1,3]. The second term is “bioactivity”, which describes various metabolic transformations, biotransformation processes, and the method of transport, interactions, and effects of substances on the body [4]. Biotransformation processes are particularly influenced by the intestinal microflora. More and more attention is being paid to its influence on the bioavailability of substances. Zhang et al. [5] indicated the influence of the intestinal microflora in terms of secreting various enzymes by which it participates in the biotransformation of foods or drugs. Biotransformation can lead to beneficial bioactive substances such as short-chain fatty acids (SCFA) and vitamins, but also other molecules like lipopolysaccharides (LPSs), amino acids (BCAAs), bile acids, and others [6]. In addition, it has been proven that the intestinal microflora can also affect the process of transporting ingredients. The influence of the microflora or its metabolites can be related in two ways: through regulation of host gene expression [5] or through substrate competition [7]. Kashyap et al. [8] proved in their study that the absorption of substances in passive transport can be higher in the absence of microflora. Although the terms “bioavailability” and “bioaccessibility” are often used interchangeably, which may cause some confusion, it should be noted that “bioavailability” is a broader concept that also includes “bioactivity”.

This manuscript aims to present the validity of using in vitro simulated digestion methods, both static and dynamic, in assessing the bioaccessibility of nutrients as well as contaminants contained in food products. Particular attention was focused on the applicability of in vitro methods in assessing the bioaccessibility of dietary supplement ingredients, the effect of diet on their bioaccessibility, chemical/pharmaceutical form, the use of encapsulation methods (product design), and the effect of dietary supplement ingredients on intestinal microflora.

## 2. Materials and Methods

Articles published between 2000 and 2024 were considered for this review, including earlier publications due to source method references. All the cited publications were in English. PubMed, Science Direct, and Scopus databases were used to search for information. The search included the words: “dietary supplements”, “food supplements”, “in vitro”, “bioaccessibility”, or “bioavailability” occurring in titles, keywords, or abstracts of publications. Searches for a combination of the words “dietary supplements” and “in vitro” yielded 1531 publications; searches for “dietary supplements” and “bioavailability” yielded 796 publications; and searches for “dietary supplements” and “bioaccessibility” yielded 64 publications. Searches for “food supplements” and “bioaccessibility/bioavailability/in vitro” resulted in a low score of thematically obtained publications. Better results were obtained by searching for “dietary supplements”. The manuscripts were then analyzed, and those related to the most relevant topic were extracted. The primary criteria for selection were as follows:

- The type of method used to study simulated digestion;
- The research material used (food, dietary supplement).

Articles that did not meet the criteria were excluded. This manuscript includes the use of simulated food digestion methods and the purpose of the review—to assess the bioavailability of dietary supplement ingredients using in vitro methods. Based on the available scientific literature, a classification of applications of methods in bioavailability assessment was established.

## 3. Digestive Mechanisms

**Figure 1:**
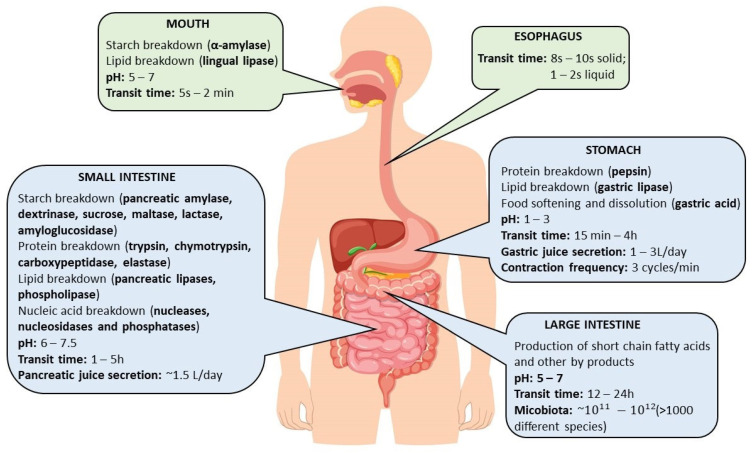
Digestive tract (prepared on the basis of literature: [17]).

The use of nutrients in biotransformation reactions, tissue reconstruction, or as substrates in energy processes is possible only after their absorption. The process of digestion is defined as the breakdown of food into simple components, the form of which allows them to be absorbed into the bloodstream and produce physiological effects [9,10]. The nature of the processes occurring during digestion allows them to be divided into three categories: mechanical, chemical, and microbial digestion. A limited number of nutrients, characterized by their small size, can directly penetrate through the intestinal wall. Membrane transport proteins from the ATP-binding cassette (ABC) family and solute carriers (SLC) are the two main groups of transporters that are present in intestinal tissues [11]. Hence, organic nutrients occurring in the form of large molecules must be converted to less complex structures that are absorbed in various sections of the gastrointestinal tract (GIT) [9,10]. The digestion process in the human digestive tract can be divided into four successive stages, taking place in the mouth (oral processing), stomach (gastric processing), small intestine (intestinal processing), large intestine, or colon (fermentation) (Figure 1) [9,10,11]. Food in the oral cavity stays for a short period of time and undergoes tasting, mixing with saliva, grinding, tearing into smaller fragments, and being brought to body temperature. To a small extent, it is subjected to the action of enzymes present in saliva, e.g., salivary amylase and mucins, which belong to glycoproteins. Obtaining the right viscosity and consistency of the food facilitates swallowing and passage through the digestive tract [10].

Then, after initial processing, the food enters the stomach. The total time required to remove nutrients from the stomach has been estimated at 4 h [12,13,14]. Camilleri et al. [12] and Metcalf et al. [15] stipulate, however, that this value varies depending on the form of the meal, its abundance of protein and fat components, and the size of the portion consumed. Despite such a long residence time of food in the stomach, the absorption process is limited or, for some nutrients, does not occur at all [16].

Most nutrient absorption occurs in the duodenum, jejunum, and ileum [18]. The duodenum is the site of the secretion of various hydrolytic enzymes. Under physiological conditions, the pancreas secretes bicarbonate, which provides the proper pH = 7 for the action of pancreatin and physiologically secreted digestive enzymes. In addition, bile acids produced in the liver, once transported to the intestine, act as emulsifiers for fats, which accelerate their breakdown by pancreatic lipase. The task of pancreatin, as well as bile acids, is to convert proteins, fats, and carbohydrates into smaller molecules that are better absorbed [18]. Conditions in the ileum and jejunum favor the activity of digestive enzymes and, due to their highly undulating surfaces, have a large area capable of absorbing small molecules [19]. The absorption process is also particularly influenced by gut microbes, which have been shown to effectively modulate the bioavailability of drugs or foods. The main function of the large intestine is to process food residues that are not digested in the small intestine. It is responsible for the absorption of water and electrolytes, the absorption of vitamins, and the production and movement of feces toward the rectum [20]. The large intestine consists of the cecum, the ascending colon, the transverse colon, the descending colon, and the sigmoid colon. The colon plays an important role in the event of a deficiency of vitamins K, E, and biotin because the bacteria residing there synthesize some vitamins via fermentation [19].

## 4. In Vitro Methods

Currently, we have many in vitro testing methods that have been developed and refined over the years. These methods aim to determine the bioaccessibility and bioavailability of nutrients from food matrices. Thanks to these methods, we are able to obtain a lot of valuable information regarding the complexity of the factors affecting their absorption. We should keep in mind that in vitro methods mainly allow us to assess the bioaccessibility of an ingredient and will not fully determine bioavailability, which has its own physiological or metabolic endpoint. In addition, in vitro tests will not take into account the nutritional status, age, genotype, or general health of the individual. We can talk about the process of bioaccessibility precisely when we measure absorption using an in vitro method to assess the degree of availability of an ingredient. Methods for determining bioaccessibility in vitro are a very good tool for assessing interactions between ingredients, the influence of factors such as pH, enzymes, the type of food used (cooking processes, technology), or the potential for absorption from different matrices, e.g., dietary supplements. Conducting simulated dietary conditions in in vitro models has been shown to be less expensive, faster, and better controlled in terms of the experimental variables studied compared to the methods used in animal studies [21]. Among the in vitro simulated digestion models used to assess bioaccessibility are solubility, dialyzability, and more advanced (dynamic) models such as TIM, for example. Cell lines like Caco-2 or HT-29 are useful in determining the bioactivity of substances [22].

### 4.1. From Simple to Advanced Methods of Simulated Digestion

The common element of all methods is to conduct in vitro digestion to simulate the human digestive system in two stages (rarely three), which are gastric and intestinal digestion. Gastric digestion uses the enzyme present in the human stomach, which is pepsin. Pepsin from the pig’s stomach is usually used. It is added to the samples before they are acidified to pH = 2 (adult) or pH = 4 (infant) [23]. This process is followed by incubation. Subsequently, samples are prepared for intestinal digestion by neutralizing to pH = 5.5–6 before adding pancreatin (pancreatic enzymes: pancreatic amylase, lipase, ribonuclease, and protease—trypsin) and bile salts (emulsifiers). After this process, the pH of the sample is brought to 6.5–7, and it is incubated again. After the digested sample is achieved, a solubility assay can be performed. The sample is centrifuged to obtain the supernatant and precipitate. The components present in the supernatant represent those that have dissolved and can be measured by various spectrometric or chromatographic methods. The result is the percentage of the soluble compound relative to the total amount of compound in the sample.

**Figure 2:**
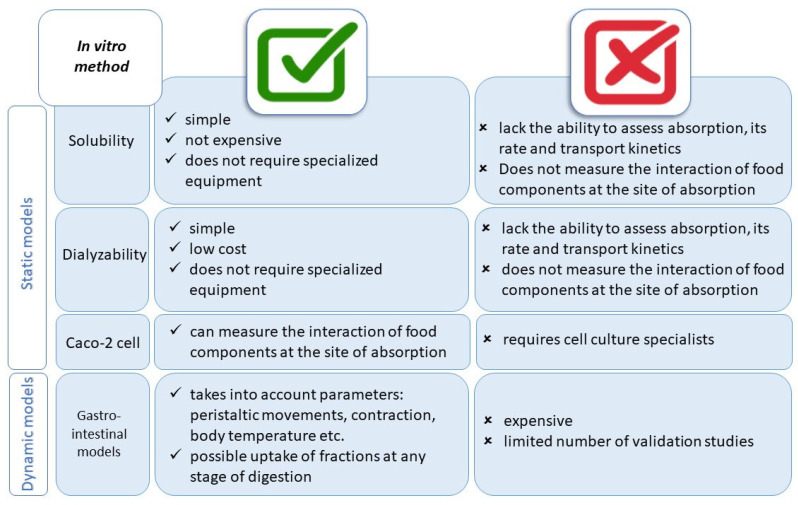
The advantages and limitations of in vitro methods’ usage [23].

Among the basic models for predicting bioaccessibility, static and dynamic models are distinguished. Figure 2 shows the advantages and disadvantages of using selected methods of simulated in vitro digestion. Static models are characterized by relative simplicity and low cost of analysis; however, they do not simulate mechanical actions. Static in vitro methods include solubility, dialyzability, and the use of cell lines. In 2014, a protocol describing a set of digestion parameters (pH, ionic composition, endogenous surfactants, and enzyme activity) for the static in vitro digestion method, INFOGEST, was published [21]. In 2019, it was improved and still functions today as the INFOGEST 2.0 protocol. A limitation of using static in vitro models is the lack of ability to use complex dynamics of the digestive process or physiological interaction with the host. The problem is the pH in the stomach, which is kept constant, which does not condition the variability of enzymes, acids, and minerals. Similarly, enzyme activity remains constant, regardless of the type of food being digested (protein, lipids, or carbohydrates). Another limitation is the intestinal phase, which is a single phase and not a chain of sequential phases of the duodenum, jejunum, and ileum (different dilutions, minerals, enzyme activity, or microbial content) [22]. Nevertheless, static in vitro methods offer a number of possibilities. So far, the INFOGEST protocol has been applied in the evaluation of the release of carotenoids and phenols [24] or proteins, lipids, and starches from foods and their modified carriers (encapsulation) [25,26]; digestion of dairy rennet gels [27]; determination of immunogenic potential of peptides from foods [28]; monitoring of enzymatic hydrolysis (using pH-stat) [29]; new polymers [30]; and digestive stability of transgenic microRNAs (genetically modified foods) [31]. The INFOGEST 2.0 protocol consists of the following steps: preparation for the determination of enzymatic activity; preparation of simulated digestive fluids: simulated salivary fluid (SSF; pH = 7), simulated gastric fluid (SGF; pH = 3) and simulated intestinal fluid (SIF; pH = 7); conducting the pH test adjustment experiment; and performing the various phases, sampling procedure, and sample treatment. In addition, the protocol lists the enzymes used in the procedure, the substances needed to determine enzymatic activity, and the necessary equipment and reagents [22]. In the INFOGEST protocol, attention was paid to the correctness of using standardized pepsin activity tests. In vitro digestion results were compared with in vivo data. An example of such studies is the publication by Rojas-Bonzi et al. [32], who studied the relationship between in vitro and in vivo starch digestion kinetics in pigs enucleated in the portal vein and fed bread with different dietary fiber contents and composition. They obtained a strong correlation between *k* values for in vitro and in vivo in their study. A second study compared the in vitro digestion results of skimmed milk proteins with in vivo results, where a porcine animal model was used. It was found that protein and peptide degradation (at the end of the gastric phase) correlated well with gastric peptides in vivo. Also, the in vitro intestinal phase correlated well with in vivo samples taken from the middle part of the jejunum [33]. Another study evaluated the bioavailability of β-cryptoxanthin from fresh orange and pasteurized orange juice in humans as well as in simulated in vitro digestion. The results of both studies gave the same answer regarding the higher bioavailability of β-cryptoxanthin from orange juice [34]. In conclusion, the method described in this INFOGEST protocol can be used to evaluate endpoints resulting from food digestion after the analysis of digestion products (e.g., peptides and amino acids, fatty acids, and simple sugars) and assess the micronutrient release from the food matrix [22].

Subsequently, in vitro methods were expanded to include the use of a dialyzability model, which was initially presented by Miller et al. [35] to determine the bioaccessibility of iron from the diet. The model assumes the measurement of low-molecular-weight solutes and presumes that solutions on both sides of the membrane strive to reach equilibrium.

Due to the development of in vitro methods for assessing nutrient bioaccessibility, the significant role of the small intestine in the absorption process has been taken into account [36]. The gut microbiota consists of several species of microorganisms, including bacteria, yeasts, and viruses [37]. The main intestinal microbes are *Firmicutes*, *Bacteroidetes*, *Actinobacteria*, *Proteobacteria*, *Fusobacteria*, and *Verrucomicrobia*, with *Firmicutes* and *Bacteroidetes* [38] representing 90% of the intestinal microbiota. *Firmicutes* consists of more than 200 different genera, such as *Lactobacillus*, *Bacillus*, *Clostridium* (95% of the *Firmicutes* group), *Enterococcus*, and Ruminicoccus. *Bacteroidetes* consist of the dominant genera *Bacteroides* and *Prevotella*, while *Actinobacteria* (less numerous) are mainly represented by the genus *Bifidobacterium* [38]. It is now established that the intestinal microflora can affect the bioavailability of a wide variety of food components [39,40], including contaminants [41]. In addition, it is involved in maintaining the proper integrity of the intestinal barrier, which is a key factor affecting the bioaccessibility of nutrients and chemicals. As a result of Yin et al.’s [42] research on the bioaccessibility of arsenic, the gut microbiota caused a speciation change in this element.

The comprehension of gastrointestinal tract processes has led to the further development of in vitro methods with advanced dynamic models. Dynamic models, on the other hand, are expensive but take into account changes in pH, mechanical actions, and fluid release over the time period studied [43]. These models include the TNO Gastro-Intestinal Model (TIM), Dynamic Gastric Model (DGM), DIDGI^®^ System, Simulator of Human Intestinal Microbial Ecosystem (SHIME), and the human stomach simulator (HGS) [44,45,46,47]. The systems’ physiological parameters taken into account are mixing, meal transport, varying pH values in place and time, the realistic secretion and composition of gastrointestinal tract fluids, and the removal of processed compounds and water. The physical model is controlled to imitate the basic conditions in vivo using computer simulation [21]. Accordingly, both static and dynamic models use the Caco-2, HT-29, or T84 cell lines in order to most accurately represent the absorption processes occurring further down the gastrointestinal tract [47,48]. These cells represent human colon tumor epithelial cells and behave similarly to cells found in the intestines [48,49]. Under the right conditions, they differentiate into mature enterocytes, forming microvilli and behaving similarly to small intestinal epithelial cells [50]. For example, Déat et al. [51], for the first time, used the dynamic TNO model (TIM) coupled with Caco-2 cell cultures to assess the bioaccessibility of lycopene and α-tocopherol from foods containing red tomatoes and sunflower oil. In turn, Shi et al. [41] used a dynamic in vitro model, SHIME, in combination with Caco-2 cell cultures for studies of *Chaenomeles speciosa*.

### 4.2. Development of In Vitro Methods

In vitro digestion methods have begun to evolve from simple to more complex models, or from 2D to 3D, using cell line cultures, primary cells, device-based cell models, organoids, organs on chips, and other 3D bioengineering approaches [52]. All these models and their modifications are intended to reproduce, as precisely as possible, an increasing range of conditions in the gastrointestinal tract. Initially, there were cell lines and simple co-culture models based on Caco-2 cells. The Caco-2 cell model has many applications, such as the assessment of the mechanisms of drugs and other food ingredients’ transport, assessment of permeability, and prediction of drugs and other bioactive substances’ absorption [53]. There are several uptake and transport mechanisms, such as transmembrane uptake and paracellular transport, which can be investigated using Caco-2 cells [54]. The Caco-2 model proves to be a very useful model for studying intestinal transporters against other in vitro digestion models. Caco-2 cells form monolayers of differentiated epithelial cells that are tightly interconnected intercellularly. Thus, they prevent the paracellular diffusion of solutes. This results in a selective barrier to designing different transport relationships in the structure for passive as well as carrier-mediated transport [55]. Other cell types that are employed as sources of cells that replicate human models of the gastrointestinal system are HT-29, T84, and LS174T [56]. To replicate the physiological functions of the digestive system as accurately as possible, several combinations of cell lines in varied proportions are now used in co-culture cell models. The use of cell models based on different cell lines is a valuable addition to assessing bioaccessibility. However, for longer experiments, the lifetime of models can be a barrier. The cause of the problem could be unanticipated cell division, a loss of cell activity, or morphological changes that have occurred.

Another valuable discovery was research based on self-organizing 3D culture systems—organoids. These systems are intended to reflect human–microbiota or diet–microbiota–human interactions as closely as possible. They constitute a widely used model to study the influence of microflora and the resulting by-products on processes such as the adhesion and infection of intestinal epithelial cells [50]. The use of organoids has some benefits as well as disadvantages. They are reliable and efficient for studying the host and microflora but costly and time-consuming [57].

Device-based models such as Transwell^®^ cocultures have also become invaluable, bridging the gap between 2D and 3D models. They are based on classic in vitro models, in which cells are cultured with test material and microflora [52]. Another device is TEER (Transendothelial Electric Resistance), which is used to study the properties of cell barriers and the effects of various compounds on them [58].

Recently, there has also been another trend toward the miniaturization of in vitro methods. Microfluidic devices (chips) have gained interest. These systems are capable of mimicking human biology in vitro on the smallest biologically acceptable scale. These devices show great applicability in many fields, featuring controlled fluid flow, integration, low wear, high throughput, and rapid analysis [59]. They represent the future for further refinement, connectivity with other in vitro models, and the potential for use in an increasingly wide range of scientific fields.

There are more and more models using cell cultures, trying to reproduce the human biological environment as closely as possible. They range from simple models to more and more complex ones, combining both. Even though it can be challenging to choose the right components to build a suitable model for study, doing so provides new possibilities for these techniques to be improved.

## 5. Application of In Vitro-Simulated Digestion Models to Assess the Bioavailability of Ingredients from Foods, Including Dietary Supplements

Currently, there are many studies using an in vitro simulated digestion model to assess the bioaccessibility of food ingredients. The most commonly studied products are dairy products [60,61], egg products [62,63], meat and seafood products [64,65], emulsified foods [66,67], fruits and vegetables [68,69], and cereal products [70,71]. Components such as elements, vitamins, macrocompounds of food (lipids, proteins), and plant substances (polyphenols, carotenoids, and chlorophylls) are among the most commonly evaluated through in vitro digestion models (Table 1). Numerous studies that focus on an integrated method for the identification of multiple components at once, as well as establishing the availability of individual components from foods, have been published [72,73,74]. Due to the often complex interactions that exist between these ingredients, it is reasonable to use simple to more complex in vitro models in assessing bioavailability. Various models of simulated in vitro digestion have been used in the available literature (Table 1).

There are many articles in the scientific literature using in vitro-simulated digestion models to assess the bioaccessibility of food components However, static in vitro digestion models are among the most commonly used, despite the contributions made to the development of dynamic models [101]. This is certainly influenced by the fact that a standardized INFOGEST model has been developed, but also by the simplicity and low cost of solubility or dialyzability models. In addition, an excellent complement is the possibility of using, in these simple models, cell cultures. Hence, the possibility of applying this tool to the evaluation of functional foods and dietary supplements, which are very popular in today’s world, is noteworthy. Table 2 presents an overview of research studies using simulated digestion methods for dietary supplements, categorized by the components. Most published studies using in vitro methods focus on assessing the bioaccessibility of ingredients contained in dietary supplements. The percentage of bioaccessibility is determined mainly from single and multi-ingredient supplements, mostly those containing vitamins and elements [102,103,104,105]. Various analytical techniques are used to assess the content of these compounds, such as high-performance liquid chromatography with fluorescence detection (HPLC-FD), high-performance liquid chromatography with diode array detection (HPLC-DAD), liquid chromatography–tandem mass spectrometry (LC-MS/MS) [106], liquid chromatography–tandem mass spectrometry with electrospray ionization (LC-ESI-MS/MS) [107], atomic absorption spectrometry (AAS) [102,108], scanning electron microscopy (SEM), fluorescence spectroscopy [109], Fourier spectroscopy (FTIR) [108,109,110], particle electrophoresis [109] inductively coupled mass spectrometry system (ICP-MS) [105], inductively coupled plasma–optical emission spectroscopy (ICP-OES) [104], and many others.

### 5.1. The Effect of a Meal on the Bioaccessibility of a Dietary Supplement Ingredient

An increasing number of studies have focused on evaluating the effect of diet on the bioaccessibility of dietary supplements’ ingredients. Jensen et al. [107] conducted a study evaluating the actual content and bioaccessibility of vitamins A, C, and folic acid from dietary supplements under both fasting and meal conditions. The authors used a static, three-step digestion model. It was found that the bioaccessibility of folic acid ranged from 24 to 100% and was higher with meal consumption than with fasting. In the case of vitamin C from dietary supplements, its bioavailability ranged between 51 and 100%, and no effect of a meal on its availability was observed. Vitamin A had a higher bioavailability from dietary supplements on an empty stomach than with meals (51–93%). Moreover, the presence of fat had an impact on vitamin A bioavailability from dietary supplements.

A similar experiment was conducted by Jensen et al. [107], who analyzed the availability of vitamin K in the gastrointestinal tract with various food matrices and from dietary supplements. The study was conducted on a three-step in vitro model using the INFOGEST 2.0 protocol. The use of an in vitro model indicated that concentration-dependent bioaccessibility may originate in the non-representative formation of mixed micelles during digestion. In turn, the size of the micelles that would accommodate the bioactive compound depends on the food, especially the fat content [120]. It has been found that the addition of a meal, the presence of vitamin D_3_, and changing the amount of bile and vitamin K did not significantly affect bioaccessibility. Nevertheless, bioaccessibility was significantly influenced by the food matrix (*p* < 0.05). The bioavailability of vitamin K ranged from 30% to 102% and was higher in canola oil and pasteurized eggs than in dietary supplements [107].

Bawiec et al. [104] estimated bioaccessibility with the single effect of dietary intake using a two-step in vitro digestion model with cellulose dialysis tubes. Selenium content was determined in dietary supplements, composed diets and fractions after digestion, respectively. There was a significant effect of the food matrix on the bioaccessibility of selenium from dietary supplements. The staple diet, characterized by a moderate amount of protein and a high content of carbohydrates and fiber, positively influenced the availability of this element in the gastrointestinal tract.

Bryszewska et al. [108] also observed the impact of the added food matrix at various stages of digestion. At the gastric stage, iron content was lower in the case of the dietary supplement consumed with bread compared to the supplement consumed alone [108].

### 5.2. The Effect of Chemical/Pharmaceutical Form on the Bioaccessibility of Dietary Supplement Ingredients

Research on the bioaccessibility of dietary supplements’ ingredients also indicates a significant impact of the chemical/pharmaceutical form of the product [102,104]. It turns out that in many cases, the organic form is better bioavailable than the inorganic form, for example, for elements. A study on the influence of the chemical form on the degree of bioaccessibility of zinc from dietary supplements was published by Ośko et al. [102]. Analyses were conducted using a two-stage static model with dialysis membranes mimicking the intestinal lumen. The zinc bioaccesibility from selected dietary supplements ranged from 1.13% to 9.38%. The in vitro digestion model allowed for the determination of the highest bioaccessible chemical form of zinc that was present in a given dietary supplement, i.e., the organic form of zinc—diglycinate. Bioaccessible zinc studies have shown the variability of this parameter, which can be influenced by many factors, such as the chemical form of the element, solubility, stability, and the amount of zinc compound in the dietary supplement, among others. It was also found that the bioaccessibility of zinc is not dependent on its dose in the product [102].

Similar research, on the influence of diet on the availability of selenium from dietary supplements, was demonstrated by Bawiec et al. [104]. With the in vitro model used, it was possible to determine the effect of diet on the bioaccessibility of selenium in different chemical and pharmaceutical forms. The highest degree of bioaccessible chemical form of selenium was obtained with dietary sodium selenate (IV) (46.18–66.10%). The highest bioaccessibility of selenium was obtained for tablets (34.7%) compared to capsules and coated tablets (*p* < 0.001). The authors have demonstrated that the bioaccessibility of selenium can be positively influenced by a diet with moderate protein and high carbohydrate and fiber content [104].

### 5.3. The Effect of the Carrier Used on the Bioaccessibility of a Dietary Supplement Ingredient

Many publications on the availability of dietary supplements’ ingredients also concern the process of substance encapsulation [108,109,110,112,113,114,116,121,122,123]. Various methods and carrier substances are used to increase their bioaccessibility in the gastrointestinal tract. The purpose of encapsulation is to protect valuable food ingredients against too rapid degradation and, in the next stage, against a lack of absorption. Among the popular substances subjected to the encapsulation method are bioactive substances such as polyphenols [109,113,114,116], coenzyme Q10 [112], vitamins [121], and elements [108,110]. Spray drying and lyophilization, coacervation, fluidized bed coating, emulsification, entrapment in liposomes, and inclusion complexation are a few techniques used to create encapsulates [122]. Nano- and microencapsulation methods with different types of carriers, such as hydrogels, emulsions (e.g., oils), solid nanolipid particles (SLN), nanostructured lipid carriers (NLCs), liposomes, or other substances, are increasingly being used [123]. The use of simple in vitro techniques in this case is proving to be a useful tool for a preliminary assessment of the behavior of a substance in a newly formulated supplement and for evaluating its availability to the gastrointestinal tract.

Grenha et al. [110] conducted research on the bioaccessibility of microencapsulated selenium in the food matrix. Due to the low solubility of the free form of selenium, its bioavailability is limited (1%). Therefore, the authors decided to use microencapsulation in their research to increase its availability, achieving an increase in bioaccessibility of up to 55%. Selenium microencapsulation was carried out using the spray-drying method with mannitol or mannitol/enteric polymer (Eduragit^®^, Evonik Health Care, Essen, Germany). It turned out that the presence of the digestion-resistant polymer resulted in the better bioaccessibility of selenium. The element was, thus, protected against interactions with other dietary factors originating from the food matrix.

In a study by Bryszewska [108], dietary supplements containing iron (in the presence of selected food products) were evaluated using an in vitro digestion model. The study also aimed to indicate the effect of microencapsulation on the bioavailability of iron from the products. The author used a two-step model to assess the degree of bioavailability of the element. The results showed that products containing encapsulated iron had higher bioavailability after digestion in the presence of a food matrix. In addition, the heat-resistant modified starch, which was used as a carrier of microcapsules, acted as a barrier, limiting the possibility of the interaction of iron ions with its complexing agents in food. Vitamin C from dietary supplements did not affect iron solubility.

Other dietary supplements’ ingredients that deserve attention are antioxidant substances. An example of research published so far is the use of the querticin microencapsulation technique. Liu et al. [115] used fatty acid (FA) and sodium caseinate (NaCas) carriers with a ligand to improve water dispersibility, storage/thermal stability, and bioaccessibility of the compound. Their use turned out to be an effective way to increase the bioaccessibility of quercetin and, at the same time, improve its dispersibility and stability.

Another bioactive compound with limited accessibility is CoQ10 due to its low solubility in aqueous solutions. In a study by Liu et al. [112] the authors encapsulated CoQ10 in whey protein nanoparticles to improve its antioxidant activity and bioavailability. The method allowed for improved antioxidant activity and increased bioavailability of CoQ10 during in vitro digestion.

Simulated digestion techniques were also used in research to increase the bioaccessibility of curcumin and epigallocatechin gallate (EGCG) following their encapsulation [109]. Strongly hydrophobic curcumin was enclosed in the hydrophobic zein cores, and weakly hydrophobic EGCG was enclosed between the zein core and the casein shell. A study conducted using a two-stage in vitro digestion model showed the high bioaccessibility of curcumin. It was also concluded that EGCG can improve the performance of zein-based curcumin nanoparticles in terms of physicochemical properties and bioaccessibility.

Another carrier in the method of encasing polyphenolic compounds was used by Toro-Uribe et al. [113]. They used lipid carriers for prostacyanidins, which ultimately increased the bioavailability and antioxidant effect compared to the non-encapsulated form. Moreover, they found that the release profile of prostacyanidin is influenced by the chemical structure and physicochemical interaction with the lipid carrier.

Another example is the study by Huang et al. [116], in which the effectiveness of the method of encapsulating resveratrol in zein/pectin core/shell nanoparticles was assessed. Bioaccessibility was evaluated using the in vitro method in a model composed of the stomach and intestine. Due to the encapsulation technique used, the bioavailability of resveratrol was higher than in the case of the free compound. The authors’ work is another example of the validity of using in vitro methods to evaluate newly developed forms of dietary supplements in which compounds with high antioxidant potential, low stability, and high importance for human health are contained.

### 5.4. Other Purposes of Studying the Impact of the Bioaccessibility of Dietary Supplement Ingredients

There are many applications of in vitro simulated digestion methods used to assess bioaccessibility. Starting from the basic estimation of the degree of availability of ingredients from dietary supplements, through assessing the impact of the diet on the bioaccessibility of the product ingredient, evaluating the impact of the chemical and pharmaceutical form on the degree of substance release, and up to the use of nano- and microencapsulation methods to assess the selection of the best carrier for the assessed substance or element. However, research focuses not only on the typical bioaccessibility of a supplement ingredient but also on the impact of its ingredients on the host’s intestinal microflora. Such research was conducted by Marzorati et al. [47], who investigated the effect of plant dietary supplements, rich in polysaccharides, on the structure, composition, and metabolism of the colonic microbial community. They used a dynamic model (SHIME) for evaluation. Based on the results, the authors concluded that long-term supplementation with mixtures of polysaccharides of plant origin had a positive effect on the intestinal microflora.

The effect of dietary supplements’ components on the intestinal microflora is extremely important. Recent studies indicate the beneficial effects of using dietary supplements containing probiotic bacteria [124]. Among the dietary supplement categories under study, prebiotic [117] as well as probiotic [118,119] supplements are the subject of research. Research focuses not only on determining the composition of these products but also, for example, on determining the transit tolerance of the effect of simulated digestion by in vitro methods on isolated probiotic bacteria [118]. The study showed a decrease in cell survival for most strains over time under the influence of gastric juice (pepsin, pH = 2). Only the strains of *Lactobacillus delbrueckii* ssp. *bulgaricus* 52, *L. acidophilus* N2, *L. acidophilus* 64, and *L. plantarum* 84 were able to survive the 90 min incubation period in pepsin solution. In contrast, no strain made it to 180 min of incubation. The presence of the analyzed strains in pancreatin solution (pH = 8), conversely, made them more resistant to the enzyme, resulting in a smaller decrease in their survival rate (30%) [118].

Similar results from survival studies of probiotic strains were published by Naissinger da Silva et al. [119]. All probiotic samples showed a reduction in the concentration of probiotic microorganisms after gastrointestinal simulation. Only some of the samples showed concentrations above 6 log CFU/g (the minimum concentration that, according to [125], is beneficial for a probiotic/dietary supplement to reach part of the ileum) in the ileum (small intestine). Unfortunately, studies show that stricter controls on the production of such products are required, as is the implementation of appropriate technologies to maintain greater viability under digestive conditions of the gastrointestinal tract.

Probiotic dietary supplements also include those containing yeast cells. Of all types of yeast, several strains of *Saccharomyces cerevisiae* have been recognized and are available for human consumption [126]. Probiotics have been shown to have health advantages whether added to food or consumed as part of a diet. [127]. Arévalo-Villena et al. [126] studied the potential of the probiotic microorganism and its use in the evaluation of *Saccharomyces* and *non-Saccharomyces* yeasts. In the study, the authors used a model of simulated gastrointestinal conditions to assess the resistance of the strains used. The study showed that *S. cerevisiae* strains are more resistant to intestinal conditions than other *Saccharomyces* strains. In turn, *S. boulardii* was not the best-rated yeast strain despite its positive control due to commercial reasons. Similar research was conducted by Gil-Rodríguez et al. [128]. They assessed the yeast’s response to physiological conditions during consumption and survival during passage through the gastrointestinal tract. About 50% of the yeasts analyzed were able to thrive at host intestinal temperatures, and about 95% of the strains could survive exposure to conditions simulating gastrointestinal transit. In Alkalbani et al.’s [129] study, the assessment of yeast probiotic products using simulated in vitro digestion methods was presented.

## 6. Conclusions

Nowadays, in vitro models are increasingly being used to study more complex matrices, such as dietary supplements. The use of in vitro models provides food and nutrition professionals with a useful, ethical, and relatively powerful tool for studying the digestibility and absorption of food components and assessing the impact of dietary intake along with the consumption of dietary supplements. Moreover, these methods provide valuable information about the content of ingredients in dietary supplements, such as macro- and microcomponents, substances of valuable bioactive nature, and probiotic strains that have a positive impact on the condition of our intestinal microbiome. This study highlights the current value of in vitro approaches that use both static and dynamic models, enriched with bacterial cultures, hence encouraging their usage in research. These models are aimed at evaluating the bioaccessibility of food ingredients, their interactions, the influence of diet, the chemical/pharmaceutical forms used, and thus the production/design methods used for dietary supplements. All these research aims are particularly important when planning menus for people struggling with deficiencies in order to supplement them more effectively. Obtaining an effective, top-quality product that meets consumer expectations and is additionally safe should be the primary goal when using these products.

## Figures and Tables

**Table 1:** Application of in vitro models to selected food ingredients.

Compound	Product	In Vitro Model	Reference
Dairy products			
Lipids and proteins	Bovine milk fat globule membrane ingredient (MFGMi) and whey–casein infant formula with added MFGMi	Solubility (INFOGEST 2.0)	[75]
Meat products			
Proteins	Chinese dry-cured hams	Solubility	[76]
Plant products			
Proteins, amino acids	Peanuts, All-Bran^®^ wheat bran cereal, pigeon peas, black beans, zein, whey protein isolate (WPI), and collagen	Solubility (INFOGEST 2.0)	[77]
Polysaccharides	3% solutions of starch, dextran, pectin, and modified citrus pectin	Solubility (INFOGEST 2.0)	[78]
Zinc	Faba bean (*Vicia Faba* L.) flour and legume fractions, finger millet (*Eleucine coracana*) flour	Dializalibity	[79,80]
Soluble dietary fiber	Whole grain noodles	Solubility	[81]
Magnesium	Mexican tortilla	Solubility	[82]
Cooper, iron, magnesium, manganese, and zinc	Purées, instant cereals, and infant formulas	Solubility (INFOGEST 2.0)	[83]
Cooper, mercury, arsenic, cadmium, lead, chromium, nickel, and zinc	Edible and medicinal plants	Solubility	[84]
Selenium and iodine	Radish *Raphanus sativus*	Solubility	[85]
Iron	Corn-masa tortillas, semisynthetic (SS) meals	Solubility, dialyzability, uptake by Caco-2 cells and/or transport assays	[86,87]
Folates	Orange juice, bread	TIM (dynamic model)	[88,89]
Carotenoids	Spinach and condensed milk (4% fat), carrots, salad meal	Solubility, uptake by Caco-2 cells	[90,91,92]
Carotenoids	Stinging nettle (*Urtica dioica* L.) in egg pasta	Solubility (INFOGEST 2.0)	[93]
Chlorophylls	Guacamole, virgin olive oil, tortellini, basil hummus, creamed spinach, vegetable pasta, green tea chocolate, avocado and kiwi juices, and pesto sauce	Solubility (INFOGEST 2.0)	[94]
Polyphenols	Cocoa, boiled and roasted peanuts; frozen sweet cherries (*Prunus avium* L.)	Solubility, uptake by Caco-2 cells	[95,96,97]
Lutein and zeaxanthin	Microalgae *Scenedesmus almeriensis*	Solubility	[98]
Lycopene and α-Tocopherol	Whole food	TIM (dynamic model) with Caco-2 cell	[51]
Oil products			
Carotenoids and chlorophylls	Oil matrix	Solubility	[99]
Other			
Calcium	Diets rich in Maillard reaction products	Solubility, dialyzability, Caco-2 cell uptake, and transport	[100]

**Table 2:** An overview of published research on the dietary supplements’ compounds that were analyzed by in vitro models.

Dietary Supplements (Compounds)	Type of In Vitro Model	Model Construction	Reference
Macrocompounds			
Mixed-polysaccharide	Dynamic model (SHIME) (stomach–small intestine–ascending–transverse and descending colon)	Stomach–small intestine–ascending–transverse and descending colon (pH regulator 5.6–5.9, 6.2–6.5, and 6.6–6.9 in the ascending colon, transverse colon, and descending colon, respectively; 72 h; 37 °C). Growth medium for the microbial inoculum consisted of a carbohydrate-based medium containing arabinogalactan (1 g/L), pectin (2 g/L), xylan (1 g/L), starch (4.2 g/L), glucose (0.4 g/L), yeast extract (3 g/L), peptone (1 g/L), mucin (4 g/L), and cysteine (0.5 g/L). The pH of the medium was 5.5.	[47]
Proteins (amino acids)	Static (INFOGEST 2.0) (stomach–small intestine)	Stomach: 11.25 mL of simulated gastric fluid (6.9 mM of KCl, 0.9 mM of KH_2_PO_4_, 25 mM of NaHCO_3_, 47.2 mM of NaCl, 0.1 mM of MgCl_2_(H_2_O)_6_, and 0.5 mM of (NH_4_)_2_CO_3_) with 2.4 mL of porcine pepsin (25,000 UI/mL and 7.5 μL of 0.3 M CaCl_2_ (pH of 3.0, 2 h, 37 °C) Small intestine: 14.3 mL of simulated intestinal fluid (6.8 mM of KCl, 0.8 mM of KH_2_PO_4_, 85 mM of NaHCO_3_, 38.4 mM of NaCl, 0.33 mM of MgCl_2_(H_2_O)_6_) with 6.5 mL of pancreatin (800 UI/mL), 2.6 mL of bile acids (160 mmol/L and 52 μL of 0.3 M CaCl_2_) (pH of 7.0, 2 h, 37 °C)	[111]
Microcompounds			
Magnesium	Static (INFOGEST 2.0) (mouth–stomach–small intestine)	Mouth: 15.1 mM of KCl, 3.7 mM of KH_2_PO_4_, 13.6 mM of NaHCO_3_, 0.15 mM of MgCl_2_(H_2_O)_6_, 0.5 mM of (NH_4_)_2_CO_3_, 1.1 mM of HCl, and 1.5 mM of CaCl_2_(H_2_O)_2_ Stomach: 6.9 mM of KCl, 0.9 mM of KH_2_PO_4_, 25 mM of NaHCO_3_, 47.2 mM of NaCl, 0.1 mM of MgCl_2_(H_2_O)_6_, and 0.5 mM of (NH_4_)_2_CO_3_ with pepsin and CaCl_2_ (2000 U/mL and 0.15 mM) (pH of 3.0, 2 h, 37 °C, 200 rpm). Small intestine: 6.8 mM of KCl, 0.8 mM of KH_2_PO_4_, 85 mM of NaHCO_3_, 38.4 mM of NaCl, and 0.33 mM of MgCl_2_(H_2_O)_6_ with pancreatin (100 U/mL and bile alts 10 mM) and CaCl_2_ (0.6 mM) (pH of 7.0, 2 h, 37 °C, 200 rpm)	[105]
Iron	Static (stomach–small intestine)	Stomach: 0.5 mL of pepsin solution (2 mg of pepsin in 0.1 M of sodium hydrogen carbonate and 0.01 M of HCl) (pH of 2.0 ± 0.1, 2 h, 37 °C) Small intestine: 0.5 mL of digestive enzyme and bile salts (0.5 mg of pancretin and 3 mg of bile salts per 1 mL) (pH of 6.8–7.0, 2 h, 37 °C)	[108]
Zinc	Static—dialyzability (stomach–small intestine)	Stomach: 0.5 mL of pepsin (pH of 2.0, 2 h, 37 °C, 140 rpm) Small intestine: 2.5 mL of intestinal solution (pH of 7.0, 2 h, 37 °C, 140 rpm) + dialysis membrane with 10 mL of PIPES	[102]
Selenium	Static—dialyzability (stomach–small intestine)	Stomach: 2 mL of 10% pepsin (pH of 2.0, 2 h, 37 °C) Small intestine: 5 mL of 0.4% pancreatin (pH of 6.5, 2 h, 37 °C) + cellulose dialysis tube	[104]
Selenium	Static (stomach–small intestine)	Stomach: 0.166 g of pepsin (pH of 2.0, 2 h, 37 °C) Small intestine: 0.034 g of pancreatin and 0.213 bile salts (pH of 5, 2 h, 37 °C) + dialysis membrane	[110]
Vitamin C	Static (mouth–stomach–small intestine)	Mouth: 5 mL of oral medium (1,7 mL of NaCl, 8 mL of urea, 15 mg of uric acid, 580 mg of α-amylase, and 50 mg of mucin in 500 mL) (pH of 7.0 ± 0.1, 5 min, 37 °C) Stomach: gastric juice (6.5 HCl, 18 mL of CaCl_2_•2H_2_O, 1 g of bovine serum albumin, 5 g of pepsin, and 3 g of mucin in 500 mL (pH of 1.5 ± 0.1, 2 h, 37 °C) Small intestine: 10 mL of duodenal juice (6.4 mL of KCl, 9 mL of CaCl_2_•2H_2_O, 1 g of bovine serum albumin, 18 g of pancreatin, and 3 g of lipase in 500 mL) (pH of 7.0 ± 0.1, 2 h) and 5 mL of bile juice (68 mL of NaHCO_3_, 10 mL of CaCl_2_•2H_2_O, 18 g of bovine serum albumin, and 60 g of bile) (pH of 7.0 ± 0.1, 2 h)	[103]
Vitamin K	Static (INFOGEST 2.0) (mouth–stomach–small intestine)	Mouth: 1 mL of saliva (2 min, 37 °C) Stomach: 2 mL of simulated gastric fluid with pepsin (2000 U/mL) and gastric lipase (60 U/mL) (pH of 3.0, 2 h, 37 °C) Small intestine: 4 mL of simulated intestinal fluid with bile (10 mM of bile salt) and pancreatin (trypsin activity of 100 U/mL) (pH of 7.0, 2 h, 37 °C)	[107]
Vitamin C, A, folic acid	Static (mouth–stomach–small intestine)	Mouth: 6 mL of saliva (pH of 6.5 ± 0.2, 5 min, 55 rpm, 37 ± 2 °C Stomach: 12 mL of gastric juice (pH of 1.5 ± 0.5, 2 h) Small intestine: 12 mL of duodenal juice and 6 mL of bile (pH of 6.0 ± 0.5, 2 h)	[106]
Other compounds			
Coenzyme Q10	Static (stomach–small intestine)	Stomach: 10 mg of pepsin (of pH 2.0, 1 h) Small intestine: 20 mg of pancreatic enzyme and 250 mg of bile salt (pH of 7.0, 2 h)	[112]
Curcumin and Epigallocatechin Gallate (EGCG)	Static (stomach–small intestine)	Stomach: 10 mg of pepsin (pH of 1.5, 37 °C, 0–1 h) Small intestine: 20 mg of trypsin enzyme and 250 mg of bile salt extract (pH of 7.0, 37 °C, 1–2 h)	[109]
Procyanidin	Static (INFOGEST 2.0) (stomach–small intestine)	Stomach: 1.6 mL ca.2500 U of porcine pepsin stock solution and 5.0 µL of 0.3 M CaCl_2_ mixed with 7.5 mL of 1.25-fold concentrated buffer solution: 6.9 mM of KCl, 0.9 mM of KH_2_PO_4_, 25 mM of NaHCO_3_, 47.2 mM of NaCl, 0.1 mM of MgCl_2_(H_2_O)_6_, 0.5 mM of (NH_4_)_2_CO_3_, and 15.6 mM of HCl; 1.6 mL of ca. 2500 U/mL porcine pepsin (pH of 3.0, 2 h at 37 °C, 100 rpm) Small intestine: 3.75 mL 800 U/mL of pancreatin solution, 1.87 mL of fresh bile, and 30 µL of 0.3 M of CaCl_2_ mixed mixed with 8.25 mL of 6.8 mM of KCl, 0.8 mM of KH_2_PO_4_, 85 mM of NaHCO_3_, 38.4 mM of NaCl, and 0.33 mM of MgCl_2_(H_2_O)_6_ and 8.4 mM of HCl (pH of 7.0, 6 h at 37 °C, 100 rpm)	[113]
Quercetin	Static (stomach–small intestine)	Stomach: 20 mL of simulated gastric fluids (2 g of NaCl and 7 mL of HCl/1 L distilled water and adjusted to pH of 1.2; then NaOH added to pH 2.5; after that, 0.064 g of pepsine added (2 h, min, 37 °C, 50 rpm) Small intestine: 187.5 mg of bile salt and 0.144 g of pancreatin (pH of 7.0, 4 h at 37 °C, 50 rpm)	[114,115]
Resveratrol	Static (stomach–small intestine)	Stomach: 15 mL of simulated gastric fluid (double-distilled water adjusted to pH of 1.2 with HCl, containing 50 mM of NaCl, and 3.2 mg/mL of pepsin) (pH of 2.5, 37 °C, 80 rpm) Small intestine: 4 mL of 12.5 mg/mL bile salt in phosphate-buffered solution (pH of 7.0, 5 mM) and 2.5 mL of 8 mg/mL pancreatin solution (pH of 7.0, 5 mM of phosphate buffer) (pH of 7.0, 4 h, 37 °C, 100 rpm)	[116]
Prebiotics	Static (INFOGEST 2.0) (mouth–stomach–small intestine)	Mouth: 5 mL of phosphate-buffered saline (PBS) mixed with 3.5 mL of simulated salivary fluid, 0.5 mL of 1500 U/mL of α-amylase, 25 μL of 0.3 M CaCl_2_, and 975 μL of distilled water (2 min, 37 °C, 150 rpm) Stomach: 7.5 mL of simulated gastric fluid, 1.6 mL of 25,000 U/mL porcine pepsin, and 5 μL of 0.3 M of CaCl_2_ and 695 μL of distilled water (pH of 2.5–3, 2 h, 100 rpm) Small intestine: 11 mL of simulated intestinal fluid, 5 mL of 800 U/mL pancreatin, 2.5 mL of 160 mM of bile salt, and 40 μL of 0.3 M of CaCl_2_ and 1.10 mL of distilled water (pH of 7, 2 h, 100 rpm)	[117]
Probiotics	Static (stomach–small intestine)	Stomach: pepsin (3 mg/mL) in sterile saline (0.5% *w*/*v*) (pH of 2.0, 3 h) Small intestine: pancreatin USP (1 mg/mL) in sterile saline (0.5% *w*/*v*) (pH of 8.0, 4 h)	[118]
Probiotics	Static (mouth–stomach–small intestine)	Mouth: 9.0 mL of peptone water (pH of 6.9, 2 min, 37 °C, 200 rpm) Stomach: 0.05 mL of pepsin (pH of 2.0, 1.5 h, 37 °C, 130 rpm) Duodenum: 0.125 mL of pancreatin, 0.125 mL of bovine bile (pH of 5.0, 20 min, 37 °C, 45 rpm) Ileum: 0.125 mL of pancreatin, 0.125 mL of bovine bile (pH of 6.5, 90 min, 37 °C, 45 rpm)	[119]

## Data Availability

No new data were created or analyzed in this study. Data sharing is not applicable to this article.
